# Right Middle Lobe Single Pulmonary Opacity With Air Bronchogram: A Problem-Solving Case Report

**DOI:** 10.7759/cureus.15204

**Published:** 2021-05-23

**Authors:** Sara Khademolhosseini, Seyedmohammad Pourshahid, Milana Zirkiyeva, Theo Trandafirescu

**Affiliations:** 1 Internal Medicine, Icahn School of Medicine at Mount Sinai, Queens Hospital Center, New York, USA

**Keywords:** maltoma, problem-solving, extranodal mzl, pulmonary maltoma, marginal zone lymphoma

## Abstract

Marginal zone lymphoma (MZL) is a relatively uncommon subtype of non-Hodgkin lymphoma consisting of extranodal, nodal, and splenic MZL. Mucosa-associated lymphoma constitutes the majority of extranodal MZL including but not limited to the stomach, lung, salivary gland, ocular adnexa, skin, and thyroid. Depending on the site of origin, maltoma may present with different symptoms.

Here we present a patient who presented to the pulmonary clinic for further evaluation of a right middle lobe consolidation. She was treated with a course of antibiotics empirically with no interval change in imaging. She underwent bronchoscopy with biopsy. Pathology was remarkable for extensive lymphoid infiltrates consisting of mixed B and T lymphocytes. Positron emission tomography (PET) CT demonstrated mild uniform uptake in consolidation with no evidence of PET avid distant metastasis. CT-guided biopsy was consistent with extranodal marginal zone lymphoma. She underwent right middle lobectomy with no complications.

As mentioned in our case, bronchoscopy is usually nondiagnostic and a lung biopsy is needed in pulmonary maltoma. Treatment is based on the tumor location and extent of the disease. Prognosis is good with an 86-95% five-year survival.

## Introduction

Marginal zone lymphoma (MZL) represents 8-12% of all B-cell non-Hodgkin lymphomas. It consists of three main subtypes: extranodal MZL (EMZL 70%), splenic MZL (SMZL 20%) and nodal MZL (NMZL 10%) [1,2]. Mucosa-associated lymphoid tissue (MALT) constitutes the majority of the EMZL and it is a clinically heterogeneous entity of low-grade B cell lymphoma that rises from different extranodal sites including stomach, lung, salivary gland, ocular adnexa, skin, and thyroid [3,4].

EMZL is known to be associated with many autoimmune situations and the underlying immunostimulation of EMZL can progress into aggressive high-grade tumors like diffuse large B cell lymphoma [5]. The presenting signs and symptoms of EMZA is usually related to the site of the tumor with cough and dyspnea as the primary symptoms associated with pulmonary associated maltoma [6].

A case of pulmonary maltoma is presented and the objective is to illustrate the diagnostic approach and treatment considering the complexity of the case.

## Case presentation

**Figure 1:**
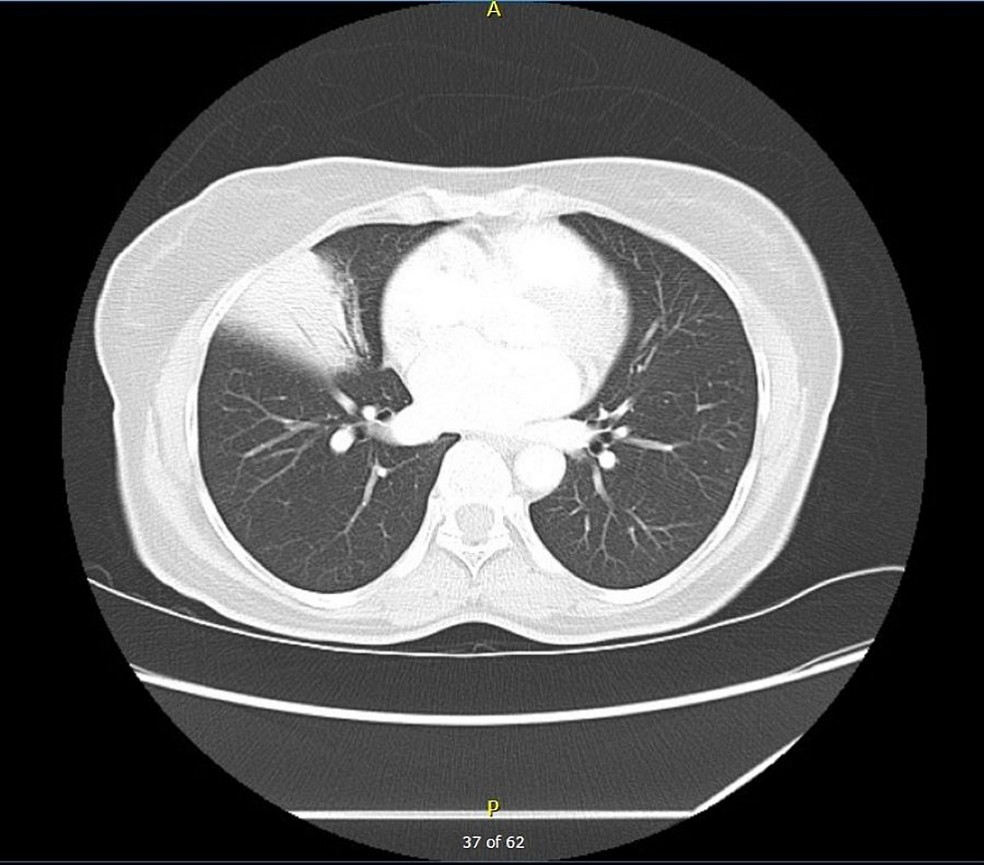
Initial CT chest was remarkable for right middle lobe opacity with air bronchogram.

A 50-year-old woman was referred to pulmonary clinic for abnormal chest CT. She underwent sigmoid colon resection for malignancy six months prior to the visit. Pathology demonstrated T3N0 moderately differentiated, invasive adenocarcinoma with clear margins. Biopsied lymph nodes were negative for malignancy. Imaging was remarkable for consolidation with air in the right middle lobe (Figure 1). Vitals were within normal limits and the only complaint was a chronic dry cough. She never smoked and the physical exam was benign.

Differential diagnosis was broad-ranging from atelectasis, reactive inflammation, infectious etiologies, and malignancy. A two-week trial of levofloxacin was started for empiric coverage of common infections.

Two months later CT showed no significant interval change. Bronchoscopy showed no significant changes on the tracheobronchial tree. Bronchoalveolar lavage showed benign bronchial epithelial cells, lymphocytes, and macrophages. Acid-fast bacilli (AFB) cultures were negative. Transbronchial biopsy revealed a bronchial wall with extensive lymphoid infiltrate consisting of a mixed population of B and T lymphocytes.

PET CT demonstrated mild uniform uptake. Differential considerations include chronic atelectasis with superimposed inflammation/infection, however, a low-grade neoplasm cannot be excluded. There was no evidence for PET avid distant metastatic disease.

Radiologic findings were not characteristic of primary pulmonary malignancy and low avidity in PET was suggestive for lymphoma, however, the biopsy revealed benign inflammatory cells with the mixed population which was against lymphoma.

Lymphoma vs slow-growing cancer was the top remaining differentials. CT-guided biopsy by interventional radiologist revealed bronchial epithelium with extensive underlying lymphocytic infiltrate consisting predominantly of small atypical B-lymphocytes (CD45+, CD20+, PAX5+, CD5-, CD10-, CD23-, BCL2+, BCL1-), with fewer interspersed small T-lymphocytes (CD45+, CD3+, CD5+).

Findings were consistent with stage I EMZL. Immunohistochemistry staining showed small neoplastic B cells positive for CD 20, CD 79a, BCL2, and CD43 with benign lymph nodes.

The patient underwent a right middle lung lobectomy with no complications. She was scheduled for follow-ups and surveillance visits.

## Discussion

MZL has an incidence rate of 1.59 per 100,000 adults. It is most common in the 6th and 7th decades of life. Maltoma is a subtype of extra nodal MZL. There are reports of maltoma in various organs. There are prior reports of maltoma in lungs but the exact prevalence is still unclear. Irrespective of the site of origin, usually maltoma is associated with a diffuse inflammatory process, thus differentiation of MZL from transformed diseases and also other indolent lymphomas is a challenge. Expert hematopathologist review is recommended to avoid overtreatment [7].

The clinical presentations of EMZL are mainly dependent on the site of the tumor [6]. Chronic cough and dyspnea is the main presentation of patients with bronchial associated MALT. Recurrent infection of B symptoms is only seen in less than 40% of patients. Lymphadenopathy is not frequently seen, and imaging reveals unilateral or bilateral pulmonary infiltrates with unremarkable mediastinum. Transbronchial biopsy is usually nondiagnostic because of the small sample size. To establish the diagnosis of pulmonary maltoma, a core needle and even surgical lung biopsy may be required [8].

Treatment is based on the tumor location and extent of the disease. For early stages of I and II extranodal disease [9], antibiotics and local approaches, like radiation and surgical resection, are preferred [10]. However, in stage III, or IV [11], chemotherapy combined with either anti-CD20 antibody rituximab or the immunomodulatory drugs is recommended.

Based on immunostimulation theory, the antibiotic regimen is the first treatment against HP-associated gastric EMZL [12]. Regression of non-gastric MZL has been reported with antibiotic therapy, although it was not specifically for pulmonary-associated maltoma [13].

Regardless of the site of involvement for EMZL, the prognosis is good, with 86-95% five-year survival [14] and median survival of more than 12 years.

## Conclusions

MZL is a relatively uncommon subtype of non-Hodgkin with different subtypes, including EMZL 70%, SMZL 20%, and NMZL 10%. Maltoma constitutes most EMZL that rises from different extranodal sites, including stomach, lung, salivary gland, ocular adnexa, skin, and thyroid. Depending on the site of origin, maltoma may present with different symptoms. Transbronchial biopsy is usually nondiagnostic. To establish the diagnosis of pulmonary maltoma, a core needle and even surgical lung biopsy may be required. Treatment is based on the tumor location and extent of the disease. Prognosis is good with 86% and 95% five-year and median survival, respectively.
